# Skewed Helper T-Cell Responses to IL-12 Family Cytokines Produced by Antigen-Presenting Cells and the Genetic Background in Behcet's Disease

**DOI:** 10.1155/2013/363859

**Published:** 2013-12-30

**Authors:** Jun Shimizu, Fumio Kaneko, Noboru Suzuki

**Affiliations:** ^1^Department of Immunology and Medicine, St. Marianna University School of Medicine, Sugao 2-16-1, Miyamae-ku, Kawasaki 216-8511, Japan; ^2^Department of Dermatology, Fukushima Medical University School of Medicine, Fukushima 960-1295, Japan

## Abstract

Behcet's disease (BD) is a multisystemic inflammatory disease and is characterized by recurrent attacks on eyes, brain, skin, and gut. There is evidence that skewed T-cell responses contributed to its pathophysiology in patients with BD. Recently, we found that Th17 cells, a new helper T (Th) cell subset, were increased in patients with BD, and both Th type 1 (Th1) and Th17 cell differentiation signaling pathways were overactivated. Several researches revealed that genetic polymorphisms in Th1/Th17 cell differentiation signaling pathways were associated with the onset of BD. Here, we summarize current findings on the Th cell subsets, their contribution to the pathogenesis of BD and the genetic backgrounds, especially in view of IL-12 family cytokine production and pattern recognition receptors of macrophages/monocytes.

## 1. Introduction 

Behcet's disease (BD) is a systemic inflammatory disease, characterized by recurrent signs and symptoms of oral aphthosis, genital ulcers, skin lesions, and uveitis. BD is not chronic inflammatory disease, but patients with BD suffer from recurrent attacks of acute and self-limiting inflammation. Repeated attacks of uveitis can lead to blindness.

The etiology of BD is largely unknown and skewed T-cell responses are associated with the development and maintenance of BD [1]. Excessive cytokine production by Th1 cells was reported using immunohistochemistry [2, 3] and intracellular cytokine staining [4, 5]. Th1 dominance was observed in BD uveitis [6] and stomatitis as well [7]. We reported excessive Th1 cell infiltration in BD skin and intestinal lesions but interleukin- (IL-) producing T cells were rarely detected [8–10]. T cells and peripheral blood mononuclear cells (PBMC) from patients with BD responded to KTH1 antigens of *Streptococcus sanguinis* in oral cavity of patients with BD and produced interferon *γ* (IFN*γ*) and IL-12 [11].

**Figure 1:**
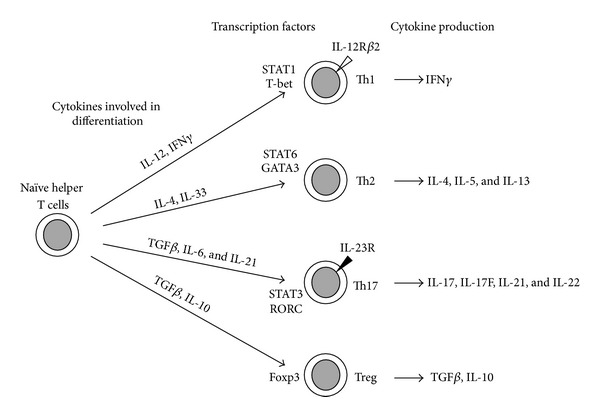
Current view of helper T (Th) cell subsets in humans [12]. Naïve Th cells differentiate into several Th cell subsets in the presence of appropriate cytokines. In response to the cytokines, the corresponding signaling molecules and transcription factors are expressed to regulate lineage commitments. Th1 and Th17 cells require IL-12 and IL-23 for their expansion, respectively. TGF*β*: transforming growth factor *β*, STAT: signal transducer and activator of transcription 3, GATA: GATA transcription factor, RORC: retinoic-acid-receptor-related orphan receptor c, and Foxp3: forkhead box P3.

Recently, Th1/Th2 paradigm was challenged by the discovery of various subsets of Th cells, such as Th17 cells and regulatory T (Treg) cells [12] (Figure 1). Researchers showed that Th cell differentiation in each subset was closely related and sometimes converted into another subset in response to environmental signals both in peripheral blood and in organs [13]. Recent studies on innate immune system suggested that antigen-presenting cells (APC) stimulated with pattern-recognition receptors (PRR) and corresponding ligands regulated Th cell differentiation by cytokine production [14].

In this review, we summarize current understanding of Th cell responses to IL-12 family cytokines produced by APC through PRR in patients with BD. We also review recent findings on the disease susceptibility genes in BD and human autoimmune diseases, which regulate immune functions.

## 2. Th1 Cells, Th17 Cells, Treg Cells, and IL-12 Family Cytokines

Th17 cells produce a number of proinflammatory cytokines, including IL-17, IL-17F, IL-21 and IL-22. IL-6, IL-21, and transforming growth factor (TGF)*β* were reported to play a role in the differentiation of Th17 cells which proliferated in the presence of IL-23 (Figure 1) [12]. Treg cells control T-cell immune responses and also need TGF*β* for their differentiation (Figure 1) [12]. TGF*β* activates Smad pathway and activated Smad protein leads to forkhead box P3 (Foxp3) expression which is a master gene of Treg cells [15]. In the presence of TGF*β*, IL-6/signal transducer and activator of transcription 3 (STAT3) signaling pathway plays a critical role in the induction of retinoic-acid-receptor-related orphan receptor c (RORC) expression which is a master gene of Th17 cells [16]. The two Th cell subsets require a common stimulation of TGF*β* for the cell differentiation, but the resultant cells show opposite immune function in the presence or absence of IL-6.

**Figure 2:**
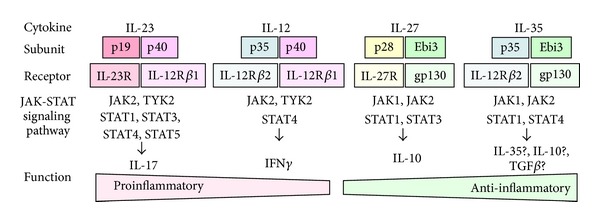
A schematic representation of IL-12 family cytokines and the corresponding receptors and JAK-STAT signaling pathways [16]. IL-12, IL-23, IL-27, and IL-35 are heterodimeric and share the subunits. The 4 cytokines require each corresponding receptor which also shares components for the function. It is thought that the 4 cytokines have overlapping but distinct effect on T cells with corresponding Janus kinase (JAK)-STAT signaling pathway. The experimental data demonstrated a functional spectrum from proinflammatory to inhibitory in Th cell differentiation. IL-12 and IL-23 are produced by activated dendritic cells and macrophages and induce inflammation through Th1 and Th17 differentiation, respectively. IL-27 is secreted from antigen-presenting cells and produces IL-10 secreting Th cells. IL-35 is mainly produced by Treg cells, amplifies IL-35 producing Th cells, and induces T-cell arrest.

As mentioned above, Th17 cells require IL-23 for the proliferation and survive, while Th1 cells require IL-12 for the differentiation (Figure 1). Recently, some researchers revealed that IL-12, IL-23, IL-27, and IL-35 are heterodimeric and share the subunits (Figure 2) and named them IL-12 family cytokines [17, 18]. IL-23 is composed of p19 and p40 subunits, IL-12 is composed of p35 and p40 subunits, IL-27 is composed of p28 and Epstein-Barr-virus-induced gene 3 (Ebi3) subunits, and IL-35 is composed of p35 and Ebi3 subunits. The 4 cytokines require each corresponding receptor which also shares components for the function (Figure 2). For example, IL-12 receptor (IL-12R) and IL-23 receptor (IL-23R) share IL-12R *β*1 subunit (IL-12R*β*1), and IL-12R and IL-35R share IL-12R *β*2 subunit (IL-12R*β*2). It is thought that the 4 cytokines have overlapping but distinct effect on T cells with corresponding Janus kinase (JAK)-STAT signaling pathway. The experimental data demonstrated a functional spectrum from proinflammatory to inhibitory in Th cell differentiation (Figure 2). IL-12 and IL-23 are produced by activated dendritic cells and macrophages and induce inflammation through Th1 and Th17 differentiation, respectively. IL-23 phosphorylates STAT1, 3, 4, and 5, but STAT4 activation, which is essential to produce IFN*γ*, is not strong compared to that in IL-12 stimulation [19]. IL-27 is secreted from APC and produces IL-10 secreting Th cells through STAT1 and 3 phosphorylation [20]. IL-35 is mainly produced by Treg cells, amplifies IL-35-producing Th cells, and induces T-cell arrest through STAT1 and 4 heterodimer's in mice [21], but the function in humans is still controversial [22].

Moreover, IL-6 and IL-11, both of which being single-molecule cytokines, need gp130 for their signal transductions in Th cell differentiation [23]. The concept of IL-12 family cytokine spectrum is simple, but physiological condition of the spectrum is supposed to be complicated. The relationship between the spectrum and TGF*β* expression remains largely unclear.

## 3. Th17 Cells, Treg Cells, and Tissue Damage

**Figure 3:**
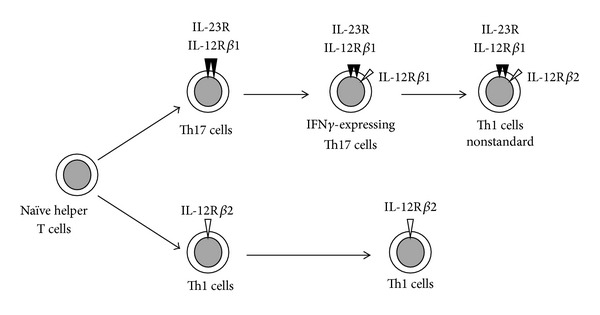
Th17 and Th1 cell differentiations and the phenotype plasticity [24, 25]. Th17 cell phenotype is not fixed *in vitro* and *in vivo* and Th17 cells can turn into IFN*γ*-expressing Th17 cells and subsequently into nonstandard Th1 cells. These two types of cells are thought to be more pathogenic and have higher affinity for inflammatory lesions than original Th17 cells.

Excessive expressions of Th17-related cytokines were found in psoriasis [26], rheumatoid arthritis [27], multiple sclerosis [28], and inflammatory bowel diseases [29]. Recently, several studies have demonstrated that Th17 cell phenotype was not fixed *in vitro* and *in vivo* and Th17 cells turned into IFN*γ* expressing Th17 cells and subsequently into nonstandard Th1 cells (Figure 3) [24, 25]. These two types of cells were thought to be more pathogenic and have higher affinity for inflammatory lesions than original Th17 cells [30–34]. IFN*γ*-expressing Th17 cells were found in several human autoimmune diseases such as Crohn's disease [30], psoriasis [31], multiple sclerosis [32], and juvenile idiopathic arthritis [33, 34].

Skewed Treg cell function was reported in many research articles of human autoimmune diseases [35]. Recent study revealed that there were differences in cell fate and functional stability between thymus-derived (t)Treg cells and periphery-induced (p)Treg cells [36]. tTreg cells had more effective functional stability, whereas pTreg cells were not stable in peripheral environment and converted into effector Th cells [37]. Epigenomic changes in Treg cells were suggested to regulate the Treg cell stability [38].

## 4. Th17 and Treg Cell Involvement in BD

**Figure 4:**
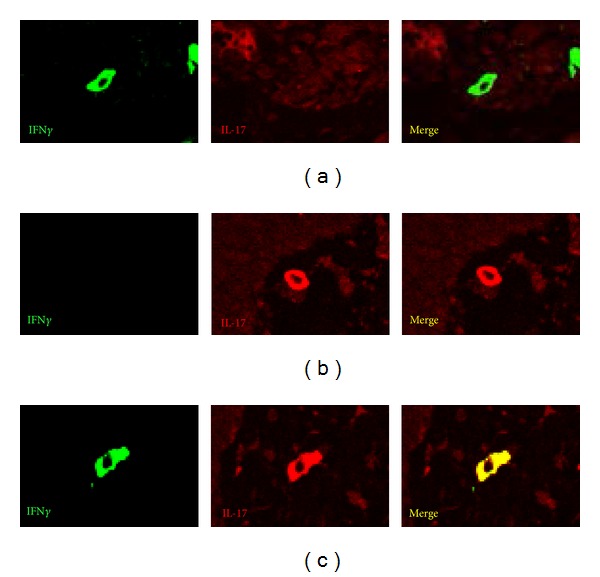
Immunofluorescence of Th1, Th17, and IFN*γ*-expressing Th17 cells in a BD skin lesion. (a) Th1 cell, (b) Th17 cell, and (c) IFN*γ*-expressing Th17 cell were simultaneously observed in one skin specimen obtained from erythema-nodosum-like lesion of BD.

It is generally thought that Th17 effector function is increased and Treg cell function is decreased in patients with BD. Overexpression of RORC mRNA [39, 40], underexpression of Foxp3 [41, 42], and high frequencies of Th17 cells [39–41, 43] were reported in patients with BD. Th17 cells were found in skin lesions [39, 40] and brain inflammatory lesions [41]. We recently reported that TGF*β*/Smad signaling pathway of mononuclear cells was overactivated in patients with BD [44]. We also reported the possibility that Th cells in patients with BD showed higher sensitivity to IL-23 and IL-12, and produced more IFN*γ* and IL-17, as compared with normal controls [40]. We observed Th1, Th17, and IFN*γ*-expressing Th17 cells simultaneously in one skin specimen obtained from erythema-nodosum-like lesion of BD (Figure 4). We speculate that both Th17 cells and Treg cells and the plasticity play a crucial role in the pathogenesis of BD.

## 5. Pathogen/Damage-Associated Molecular Patterns (PAMP/DAMP) and Toll-Like Receptors (TLR)

Phagocytes were thought to be activated by various pathogens and pathogen-derived antigens in innate immune responses. Recent studies provided evidence for the existence of specific receptors on the phagocytes against the microbial antigens where they were named pattern-recognition receptors (PRR). The receptors are not rearranged even with adaptive immune system and recognize bacterial and viral pieces, known as pathogen-associated molecular patterns (PAMP). PAMP are indispensable parts of the microbes, such as lipopolysaccaride (LPS), peptidoglycan, bacterial DNA/heat shock proteins (HSP) and viral DNA/RNA [45]. Interaction between PRR and PAMP and subsequent induction of innate immune function are highly conserved among species [46]. Phagocytes with PRR recognition produced proinflammatory cytokines and upregulated major histocompatibility complex (MHC) proteins for the promotion of adaptive immune function [47].

Toll-like receptors (TLR) are transmembrane glycoproteins and called membrane-associated PRR. Ten functional human TLR have been identified [48]. TLR1, TLR2, TLR4, TLR5, and TLR6 were expressed on phagocyte cell surfaces and TLR3, TLR7, TLR8, and TLR9 localized within intracellular vesicles. It was shown that cell surface TLR recognized cell membrane-type PAMP, such as LPS and peptidoglycan, and intracellular TLR recognized nucleic-acid-type PAMP [49].

**Figure 5:**
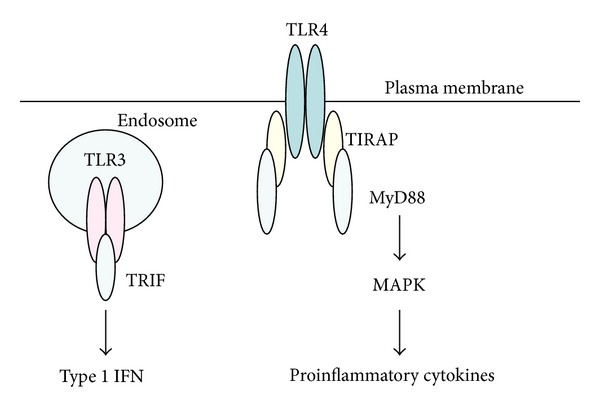
Two major TLR signaling pathways [48]. With TLR stimulation, except TLR3, APC produced proinflammatory cytokines through MyD88 and activated mitogen-activated protein kinases (MAPK). APC produced type 1 IFN by utilizing of TRIF through TLR3 stimulation, an intracellular TLR. TIRAP: Toll/interleukin 1 receptor (TIR) domain containing adaptor protein, MyD88: myeloid differentiation primary response protein 88, TRIF: TIR domain-containing adaptor-inducing IFN*β*, MAPK: mitogen-activated protein kinases, and IFN: interferon.

TLR also recognize endogenous damage-associated molecular patterns (DAMP) which are secreted from severe damaged host cells caused by any environmental stress, such as microbial infection or injury. Self-DNA/RNA, high-mobility group box1 (HMGB1), a DNA-binding nuclear protein, and self-HSP are included in the DAMP. These molecules were reported to be rapidly released following unprogrammed cell death and activate PRR-expressing cells similar to the PAMP [50]. Major TLR, PAMP, and DAMP were summarized in Table 1. In PAMP, bacterial lipopeptides, HSP, and LPS were recognized by TLR1/TLR2/TLR6, TLR2/TLR4, and TLR4 with CD14, respectively [46]. Similar mechanisms were found in DAMP with self-lipoproteins, self-HSP, and HMGB1. Two major TLR signaling pathways were demonstrated, namely, myeloid differentiation primary response protein (MyD)88-dependent pathway and Toll/interleukin receptor 1 (TIR) domain-containing adaptor-inducing IFN*β* (TRIF)-dependent pathway (Figure 5). With TLR stimulation, except TLR3, APC produced proinflammatory cytokines through MyD88 and activated mitogen-activated protein kinases (MAPK). APC produced type 1 IFN by utilizing of TRIF through TLR3 stimulation, an intracellular TLR [46].

## 6. Th Cell Differentiation through TLR Stimulation

Dendritic cells stimulated with TLR2 and TLR4 ligands produced IL-12 and IL-23 [51, 52]. APC secreted IL-27 through TLR3 and TLR4 signaling [53–55] and type 1 IFN enhanced the expression [53, 54]. It was found that each IL-12 family subunit (Figure 2) had an expression pattern in APC through TLR4 stimulation [55]. For example, APC expressed p19 during early phase for a short time and produced p35 and p40 continuously in later phase. P28 acted as an intermediary between them. These data suggest that TLR stimulation may play a role in autocrine activation of APC by type 1 IFN induction (Figure 5) and the APC regulate T-cell differentiation though IL-12 family cytokines in a time-dependent manner.

Th cells are suggested to express TLR [14]. T-cell receptor (TCR) stimulation activates T cells by phosphorylation of extracellular signal-regulated kinases (ERK)1/2, both of which are subsets of MAPK family. TLR2 costimulation to the human TCR signaling promoted the phosphorylation and directly modulated the T-cell differentiation [56]. Several researchers demonstrated that TLR2 signaling without APC led to the induction of not only Th1 [57–59] and Th17 [60] cells but also Treg cells [57] in mouse experiments. Human naïve and Treg cells converted into Th17 cells with stimulation of TLR ligands [61]. In human infectious disease, TLR2 receptor on Th cells of patients with tuberculosis was overexpressed and its stimulation caused a marked activation of the cells [62]. In contrast, underexpression of TLR2 on Th cells and lower secretion of IFN*γ* by TLR stimulation were observed in patients with filarial infection [63]. A possibility was considered that the repeated antigen exposure may explain the discrepancy [14].

Experimental approaches demonstrated various aspects of the relationship between TCR and TLR4 stimulation. TLR4 co-stimulation inhibited ERK1/2 phosphorylation of Th cells in mice [64] and TCR signaling with a pretreatment of LPS decreased activated MAPK [58]. TLR4 co-stimulation did not directly regulate Th cell differentiation, but selective deletion of TLR4 in Th cells decreased IFN*γ* and IL-17 production at experimentally inflammatory sites [65].

These results suggest a need to assess the molecular relationship between MAPK/ERK and JAK/STAT signaling pathways in Th cell differentiation under both physiological and pathological conditions.

## 7. Possible Effects of HSP on Th Cell Activation as Both PAMP and DAMP

HSP are highly conserved and ubiquitously expressed proteins and function as an intracellular chaperonin for other proteins. An HSP was found as a remarkably increased factor in *Drosophila* salivary glands with “heat shock” in the first study. After numerous studies, subgroups of HSP were named for their molecular weights and subdivided into two major functional systems. HSP60-HSP10 system assisted the adequate protein folding and HSP70-HSP40 system was involved in the stability of cytosol peptides [66]. Significant sequence homology is found between mammalian and microbial HSP. For example, mycobacterial and streptococcal HSP65 have more than 90% homology, and mycobacterial HSP65 and human HSP60 have 42% homology [67].

It was suggested that HSP were secreted from both microbes and necrotic cells and were recognized by TLR2 and TLR4 [46]. In several studies, HSP were categorized into both PAMP and DAMP (Table 1) [50, 68]. Certainly, clinical studies demonstrated that HSP accumulation was promoted in the lesions of several human autoimmune diseases [69–72]. HSP peptide-specific T cells were found in patients with type 1 diabetes [73, 74], rheumatoid arthritis [75], and juvenile idiopathic arthritis [76]. Several experimental model studies of autoimmunity reported protective effects of HSP peptide by deletion of peptide specific T cells [77]. In fact, oral administration of an HSP peptide successfully increased Treg cells [75] and reduced disease activity in patients with rheumatoid arthritis [78].

## 8. TLR and HSP Involvement in BD

Clinical studies demonstrated that both TLR and HSP expressions increased in patients with BD. Elevated gene expressions of TLR2 and TLR4 were found in peripheral blood monocytes [79], PBMC [80], polymorphonuclear leukocytes [80], bronchoalveolar lavage leukocytes [81], and oral mucosa [82] in patients with BD compared to normal controls. TLR2-and TLR4-positive cells in buccal lesions [83] and TLR6-positive polymorphonuclear leukocytes cultured with HSP60 [84] were significantly increased in patients with BD.

Several researchers observed massive expressions of HSP60 in BD skin [85] and oral ulcer lesions [86, 87]. HSP60 was expressed more diffusely [87] and intensely [85, 87] in BD lesions than those in other types of inflammation, such as oral lichen planus and recurrent aphthous stomatitis. Excessive T- and B-cell responses to major four peptides of *Mycobacterium tuberculosis* HSP65 and human counterparts of HSP60 were observed in patients with BD who lived in Europe, Far-Eastern Asia, and Middle East [10, 88–90].

We have found that TLR2 and TLR4 mRNA were expressed on ileocaecal ulcer lesions of BD, but less on unaffected sites of BD and on Crohn's disease lesions. IL-12 producing TLR2 positive macrophages located neighboring to T cells and HSP60 was expressed on the same region of the intestinal lesions [8, 9]. C-C-type chemokine receptor (CCR)5 and macrophage inflammatory protein (MIP)1*β*, a Th1 related chemokine receptor and its ligand, were detected in the intestinal lesions of BD and CCR5/MIP1*β* interaction was thought to play a role in the migration of activated Th1 cells [9]. Moreover, we have reported that Th cells yielded proliferative responses to human HSP60 peptide in Japanese BD patients by a TCR V*β* gene restricted antigen-driven process [90]. We suggest that TLR/HSP60 interactions induce destructive Th1-type responses at the intestinal lesion in patients with BD [91].

## 9. Genetic Variations of IL-12 Family Genes in BD and Human Autoimmune Diseases

**Figure 6:**
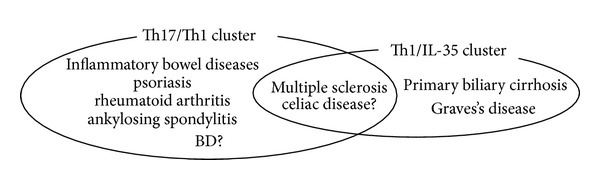
IL-12-family-cytokine-related genetic polymorphisms were found to be associated with several human immune diseases [44]. Th17/Th1 cluster was related to the polymorphisms of IL-23R and IL-12B and affiliated with inflammatory bowel diseases, psoriasis, ankylosing spondylitis, and rheumatoid arthritis. Th1/IL-35 cluster was related to the polymorphisms of IL-12A and IL-12R*β*2, and affiliated with primary biliary cirrhosis and Graves's disease. Several studies suggest that celiac disease and multiple sclerosis show both clusters' polymorphisms. Several Genome-Wide Association Studies identified IL-23R-IL12RB2, STAT4, and IL-17A as BD susceptibility genes and indicated a possibility that BD was including in Th17/Th1 cluster.

Detailed analysis of comorbidity in dozens of human autoimmune diseases revealed the importance of treating the diseases as one group and suggested that there were several common etiopathologies among the diseases [92]. In the past decade, genetic clustering in the human autoimmune diseases has progressed with Genome-Wide Association studies (GWAS) to invest underlying genetic factors. Particulary, there have been noteworthy advances in the research of genetic variants in IL-12-family-related genes, which have shown major two subclusters, namely, Th17/Th1 cluster and Th1/IL-35 cluster (Figure 6) [93]. Th17/Th1 cluster was related to the polymorphisms of IL-23R and IL-12B and affiliated with inflammatory bowel diseases [94], psoriasis [95], ankylosing spondylitis [96], and rheumatoid arthritis [97]. Th1/IL-35 cluster was related to the polymorphisms of IL-12A and IL-12R*β*2 and affiliated with primary biliary cirrhosis [98] and Graves' disease (Figure 6) [99]. Several studies suggest that celiac disease [100] and multiple sclerosis [101] show both clusters' polymorphisms (Figure 6).

A decade of GWAS was conducted for BD in Turkey [102–104], Japan [105, 106], China [107], Iran [108], and Korea [109]. Human leukocyte antigen (HLA)-B51 is the most strongly associated risk factor for BD by a meta-analysis of case control genetic association studies [110] and the GWAS data support the result [102, 103, 106]. Recent two major studies [103, 105] identified MHC class I locus, IL-10, and IL-23R-IL12RB2 as BD susceptibility genes. IL-10 is an inhibitory cytokine to both T cells and APC [111], and secreted from T cells under IL-27 stimulation, as it was previously mentioned in Section 2. IL-10 production of healthy donors' PBMC with a BD-associated allele was significantly decreased compared to that without the allele in the presence of LPS [103]. Other several studies reported that, adding to IL-10 [108] and IL-23R-IL12RB2 [108, 109], STAT4 [107, 109] and IL-17A [109] genes were associated with BD. These data indicated a possibility that BD was included in Th17/Th1 cluster according to the above-mentioned clustering analysis. The IL-12 family cytokine gene polymorphisms suggest that the function of each IL-12 family cytokine subunit molecule needs to be reinvestigated based on the clustering analysis in patients with BD.

## 10. Genetic Variations of TLR and HSP in BD and Human Immune Diseases

Researchers mentioned that TLR gene polymorphisms were associated with several allergic and inflammatory diseases [112–116]. Skewed monocyte and mononuclear cell responses in cytokine production against microbe extracts were found in atopic dermatitis and asthma patients with a TLR2 [112] and a TLR4 [113] polymorphisms, respectively.

Several TLR gene polymorphism studies in patients with BD demonstrated no association with susceptibility to BD [117–124]. Recently, a targeted resequencing study was undertaken to detect rare genetic variants and, adding to IL-23R, TLR4 and nucleotide-binding oligomerization domain 2 (NOD2) genes, the latter of which was an intracellular PRR, were found to be associated with BD [125]. MyD88 adaptor-like protein (Mal), also known as TIR domain-containing adaptor protein (TIRAP, Figure 5), polymorphism was suggested to be associated with BD in UK [83]. TLR2 and TLR4 use TIRAP as an additional adaptor to recruit MyD88 [46]. The two studies offered new approaches for identifying BD susceptibility gene. Moreover, Killer cell lectin-like receptor subfamily C, member 4 (KLRC4) gene, a natural killer cell receptor, and endoplasmic reticular aminopeptidase 1 (ERAP1) gene, a major immunoregulatory molecule by peptide trimming inside the reticulum, were identified as BD susceptibility genes [102]. These analyses of gene polymorphisms in BD, with the high susceptibility of HLA-B51, indicated the importance of innate immune function as an effective therapeutic target in patients with BD. In fact, inhibitors of tumor necrosis factor *α*, a downstream effector cytokine of MAPK signaling pathway in APC with TLR4 stimulation, remarkably ameliorated clinical symptoms in patients with BD [126, 127].

It was reported that HSP and the promoter gene polymorphisms were associated with Crohn's disease [128], bacterial sepsis [129], and multiple organ dysfunction after severe trauma [130]. HSP genes may serve as important factors for the detection of BD susceptibility gene.

## 11. Conclusions 

We reviewed here current concept in Th cell differentiation and the functional/genetic contribution of the cells to the pathogenesis of BD. Skewed IL-12 family cytokine responses and related genetic variants were suggested to play a crucial role in the pathophysiological conditions in BD. Interestingly, dysregulation of Th17/Th1 cells and genetic variation in IL-12 gene family were found in several human autoimmune diseases. The existence of genetic variants both in innate and adaptive immune responses suggests that it is important to understand the molecular mechanical differences in the Th cell responses of BD between with and without APC of the patients with BD.

## Figures and Tables

**Table 1:** TLR and corresponding PAMP and DAMP [46, 50].

TLR	PAMP	DAMP
TLR1	Bacterial lipopeptide	
TLR2	HSP (mycobacteria, Chlamydia), LPS, bacterial lipopeptide, peptidoglycan	HSP, HMGB1, and lipoprotein
TLR3	Viral RNA	Self-RNA
TLR4	HSP (mycobacteria, Chlamydia), LPS	HSP60, HSP70, HMGB1, and lipoprotein
TLR6	Bacterial lipopeptide	
TLR7	Viral and bacterial RNA	Chromatin and ribonucleoprotein, self-DNA
TLR9	Viral, bacterial and parasitic DNA	HSP, chromatin and ribonucleoprotein, and self-DNA

TLR: Toll-like receptors; DAMP: damage-associated molecular patterns; PAMP: pathogen-associated molecular patterns; HSP: heat shock proteins; HMGB1: high-mobility group box1; LPS: lipopolysaccharide.
